# Bias induced ferromagnetism and half-metallicity in graphene nano-ribbons

**DOI:** 10.1038/s41598-017-17091-w

**Published:** 2017-12-06

**Authors:** Rita Maji, Joydeep Bhattacharjee

**Affiliations:** School of Physical Sciences, National Institute of Science Education and Research, Homi Bhava National Institute, Jatani, P.O, Khurda, Odisha 752050 India

## Abstract

Towards spin selective electronics made of three coordinated carbon atoms, here we computationally propose robust and reversibly bias driven evolution of pristine undoped graphene nano-ribbons(GNR) into ferromagnetic-semiconductor, metal or a half metal, irrespective of their edge configurations. The evolution is a result of a rare ferromagnetic(FM) order emerging among nearest neighbouring(n-n) sites, in positively biased regions in their in-homogeneous bias unit-cells, in attempt to cooperatively minimise on-site Coulomb repulsion and kinetic energy, while maximising localization of electrons at the positively biased sites. The phenomenon appears to be a general property of in-homogeneously biased Coulomb correlated bipartite systems. Consequences are particularly rich in zigzag edged graphene nano-ribbons(ZGNR) due to the contest of bias driven n-n FM order and the inter-edge antiferromagnetic order inherent to ZGNRs, leading to systematic closing of gap for one of the spins, amounting to bias controlled unmissable opening of window for FM-semiconducting and half-metallic transport.

## Introduction

Sheets, ribbons and tubes made of three coordinated *sp* ^2^ hybridized carbon(C) atoms can be semiconducting or metallic^1,2^, depending on their shape, size and edge configuration. They have been thus long anticipated to constitute a framework for carbon based electronic circuitry at nano-scale^3^. 2*p*_*z*_ electrons of these carbon atoms, if rendered unpaired due to lack of coordination, like at defects or edges, are source of local magnetic moments^4,5^, and ferrimagnetism in their vicinity. Tuning magnetism of these electrons to add spin-selectivity to carbon based circuit elements, has been an active area of research^6^ in the last two decades or so. A large variety of proposals and demonstrations made in this direction based on structural^7–10^, physical^4,11–15^, and chemical^16–22^, functionalization, have promised the possibility of magnetic semiconductors and half-metal^13,23–26^, primarily in zigzag edged graphene segments, ribbons and tubes^4,27,28^. Realization of such proposals into commercially viable devices is challenged by the stringent requirement of precise control over their shape, size, and edge configurations.

In undoped bipartite systems magnetism is known to arise either at strong coupling^29–31^, where the strength of Coulomb correlation(U) is much higher than kinetic energy, leading to n-n anti-ferromagnetic(AFM) order, or at moderate coupling due to in-equivalence of the two sub-structures^32^, leading to n-n ferrimagntic(FeM) order. The latter leads to non-zero magnetic moment if the two sub-structures are globally in-equivalent^5^. Majority of proposals referred above are in this category, where the in-equivalence can be due to a host of reasons, like, vacancy defects^21^, substitutional doping^20^, adsorption at sites^33^, application of transverse electric field^13,14^. On the other hand, nearest neighbour ferro-magnetic (FM) ordering, which we will refer as FM_*n* −*n*_, is rare in bipartite systems and proposed only upon doping by hole or electrons^34,35^. Description of magnetism sourced at Coulomb correlation among itinerant electrons, as derived within the framework of Hubbard model^36^ suggests primarily two classes of mechanisms to rationalize FM_*n* −*n*_ ordering in bipartite systems upon deviation from half-filling^37^. With *U* → ∞, it was shown by Nagaoka^38,39^ that upon doping by a single hole the ground state will have FM ordering in attempt to reduce the kinetic energy of the hole, while avoiding occupancy of a site by more than one electron. However, Nagaoka-FM has been argued to be not relevant to three coordinated systems^31^, since the loops connecting the n-n sites should not pass through more than four sites for Nagaoka-FM to sustain. In the other class of mechanisms, FM ordering is proposed to be exchange driven, but require a flat or nearly flat band^40,41^, at Fermi energy to accommodate electrons emptied from the doubly occupied states without causing any or much any increase in kinetic energy. Itinerant electrons have been also argued^42^ to propagate exchange interaction between local moments due to flat bands. An approximate meeting ground of the two pathways lead to the Stoner criteria^43^, which argues that a high *U* and non-zero density of states (DOS) at Fermi energy is necessary for the unequal number of electrons with the two spins to be energetically favorable. Flat-band based mechanisms, or more generally the Stoner Criteria, for FM_*n* −*n*_, is thus supported only by the doped armchair edged graphene nano-ribbons(AGNR) on account of the flat or nearly flat bands which are located below and above the valence and conduction band edges and represent *pi*-bonds parallel to the ribbon edges. In zigzag edged graphene nano-ribbons(ZGNR), FM_*n* −*n*_ has been proposed^44^ to be possible with topological line defects owing to their non-bipartite nature.

Based on mean-field and ab-initio computation of spin resolved electronic structure, in this work we suggests an alternate approach to manipulate magnetism in graphene nano-ribbons(GNR), wherein, any GNR irrespective of its edge configuration, can be controllably as well as reversibly, turned into a FM semiconductor or metal and a half-metal, exclusively through spatially in-homogeneous biasing. Central to this approach is the robust emergence of FM_*n* −*n*_ in the positively biased regions of the unit-cell, which we argue below to be a general property of in-homogeneously biased bipartite systems, arising primarily as a means to avoid increase of on-site Coulomb repulsion and kinetic energy while maximizing response to the external bias. This work points to a new class of mechanism for emergence of FM_*n* −*n*_ order in bipartite systems in general at half filling, which is a clear departure from the body of work reported over the years on manipulation of magnetism. We demonstrate in the following the onset of FM_*n* −*n*_ accompanied by lifting of spin degeneracy and consequent opening of window for ferromagnetic and half-metallic transport, in a representative variety of AGNRs, ZGNRs and also in a minimal model system.

## Results and Discussion

**Figure 1:**
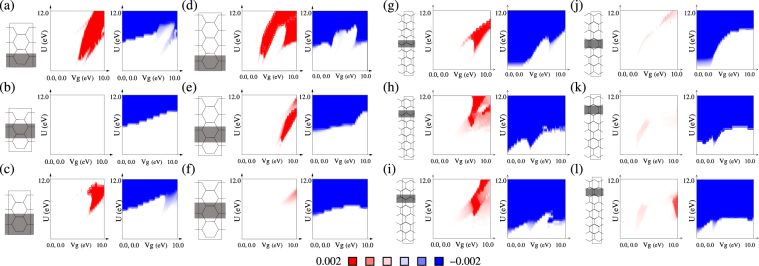
spin-correlation (positive & negative) plot as function of *U* & *V* ^*g*^ for different bias coverages: AGNR(N = 6) (**a**–**c**); AGNR(N = 8) (**d**–**f**); ZGNR(N = 18) (**g**–**i**); ZGNR(N = 16) (**j**–**l**). The degree of influence of width and location of biased region on the range of *V* ^*g*^ and *U* in which FM_*n* −*n*_ would emerge, differs from ZGNRs to AGNRs. The difference can be attributed to the presence of weak but non-zero n-n FeM order in ZGNRs away from the edges. Wider biased region imply weaker localization, and thereby, weak spin separation. These results imply significance of localization and inherent magnetic order on emergence of FM_*n* −*n*_.

We calculate spin polarized electronic structure of non-uniformly biased AGNR and ZGNR unit-cells shown in Fig. 1, first using the mean-field approximation of Hubbard model^31^ as a function of bias potential *V* ^*g*^ and on-site Coulomb repulsion *U*, and then compare with density functional theory(DFT)^45^ based first principles calculation.

To probe the nature of magnetic ordering we calculate the average n-n spin correlation as:

1 $$S=\frac{1}{{N}_{s}}\sum _{i}^{{N}_{s}}\frac{1}{{{\rm{nn}}}_{i}}\sum _{j}^{{{\rm{nn}}}_{i}}{S}_{i}{S}_{j},$$

where *N*_*s*_ is the number of sites per unit-cell, *nn*_*i*_ the number of n-n sites around the *i*-th site, and *S*_*i*_ = 〈*n*_*i*,*σ*_〉 − 〈*n*_*i*,*σ*′_〉, with 〈*n*_*i*,*σ*(*σ*′)_〉 being the population of electron with spin-*σ*(*σ*′) at the *i*-th site due to the occupied states calculated using the mean-field approximation of Hubbard model. Positive and negative values of *S* plotted in Fig. 1 imply existence of FM and AFM or FeM ordering respectively. Existence of both thus imply spatial separation between FM and FeM ordering.

### Negative spin correlation

For AGNRs, Fig. 1(a–f) imply rapid consolidation of AFM(FeM) ordering above *U*~2|*t*| with zero(positive) *V* ^*g*^. For *V* ^*g*^ = 0 this is reminiscent of Mott transition^46^ at half-filling (*n *= 1) in bipartite lattices. The trend that with increasing *V* ^*g*^ the transition from non-magnetic to the FeM ground state happens with increasing *U*, is similar to that observed in case of non-magnetic to AFM transition in bipartite lattices with increasing deviation from half-filling, and is understood in terms of the additional correlation required to dominate over the kinetic energy of the excess charges. The similarity in trend is expected since with non-zero *V* ^*g*^ the biased and unbiased regions both deviate locally from half-filling. With *U *> 0 at *v* ^*g*^ = 0, ZGNRs expectedly show n-n FeM ordering and AFM ordering globally between the two substructures. With increasing *V* ^*g*^, quenching of magnetic ordering in ZGNRs below an increasing threshold of *U* can be understood as the dominance of positive bias over on-site Coulomb correlation, leading to occupation of biased sites by electrons with both the spins.

### Positive spin correlation

**Figure 2:**
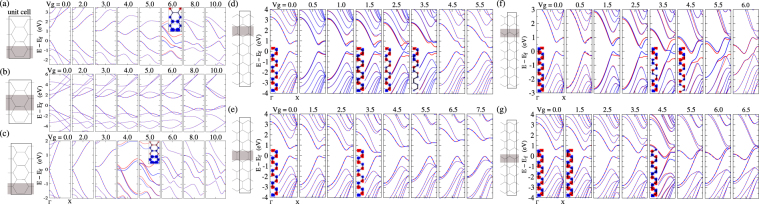
Band structure and spin density (inset) plot based on mean-field Hubbard model for *U* = 4.0 eV for different bias coverage: AGNR(N = 6) (**a**,**b**); AGNR(N = 9) (**c**); ZGNR(N = 16) (**d**,**e**: bias coverage of transverse C-C bonds); ZGNR(N = 18) (**f**,**g**: bias coverage of zig-zag C-C bonds). Emergence of FM semiconducting and half-metallic phases are more robust and prominent in case of ZGNR due to the contest between emergent FM_*n* −*n*_ order and the inter-edge AFM order inherent to ZGNRs.

Emergence of FM_*n* −*n*_ ordering is marked by the positive spin correlation [Fig. 1] and the associated lifting of spin degeneracy of the band-structures [Fig. 2] over a range of *V* ^*g*^ with *U* moderate and higher. FM ordering quenches rapidly in AGNRs [Fig. 1(a–f)] as the biased region moves away from the edges or are widened. Similarly in ZGNRs, positive spin correlation is much prominent if the biased region cover zigzag chains of carbon atoms parallel to the edges. Notably, an FM phase of generalized Nagaoka type is known to occur in the mean-field phase diagram of cubic bipartite lattice^31^ at deviation from half-filling. Although Nagaoka may not be feasible^31^ in three coordinated bipartite lattices, it is beyond the scope of this work to comment on whether it will be effective on the background of increased correlation due to confinement. However, the trend that the onset of the FM_*n* −*n*_ order is more prominent if the biased region is narrow and located closer to the ribbon edges, clearly suggest that localization of electrons, and consequently the enhanced Coulomb correlation, are likely the key associated factors leading to FM_*n* −*n*_. Notably, in case of ZGNRs, if the biased sites cover zigzag(transverse) C-C bonds then the spin at those FM_*n* −*n*_ ordered sites would prefer to be FM(AFM) ordered with sites at both the edges, mediated by the inherent n-n FeM order prevalent outside the biased region. Thus in case of biased zigzag sites, mixing of FM_*n* −*n*_ ordered state with the dominant spin, and the nearest localized edge state, can stabilize both, leading to relative ease in occurrence of FM_*n* −*n*_ order compared to that in case of biased transverse sites. This is consistent with less positive spin-correlation [Fig. 1(j–l)] in case of biased C-C transverse bonds.

### Band-structure

FM_*n* −*n*_ order at the positively biased sites inherently implies lifting of spin-degeneracy since it consolidates one of the spins in the biased region. Emergence of the FM-semiconducting, FM-metallic and half-metallic phases are thus naturally expected as consequences of FM_*n* −*n*_. However, for AGNRs, onset of FM_*n* −*n*_ order is preceded by shrinking of band-gap and direct to indirect transition. With increasing *V* ^*g*^, the bands representing the bonding and anti-bonding orbitals of the biased region will come down in energy, while their counterparts associated with the edge farthest from the biased region will have least change in energies, resulting into a net reduction in band gap, as observed. Thus the conduction(valence) band edges can be spatially located at the positively(zero) biased regions of the unit-cell. In such a scenario a direct to indirect transition is expected, since the conduction and valence band edges, which are at different *V* ^*g*^, tend to have same total energies as the band-gap shrinks, implying that their kinetic energies must be different. With increasing *V* ^*g*^, further lowering of the conduction band edge leads to complete closure of the indirect band gap. The AGNRs are thus most likely to be normal or FM-metal upon emergence of FM_*n* −*n*_ order [Fig. 2(a,c)], although a small window for half-metallic transport can open up serendipitously. As the biased region moves towards the bulk(middle) of ribbons, lowering of kinetic energy increasingly compensates Coulomb correlation, leading largely to non-magnetic ground state and spin-degenerate band structure [Fig. 2(b)].

**Figure 3:**
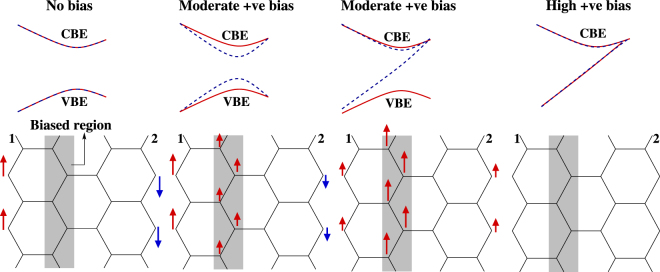
Schematic view of band structure (above) & spin density (*S*_*i*_) (below). The edges and the biased regions are mediated by the n-n FeM ordering, owing to which, the FM_*n* −*n*_ ordering with spin-1(↑) in the biased region induces preference for the same spin at the edges, leading to suppression of the edge state at edge-2 with spin-2 (↓). The evolution of the conduction and valence band edges (CBE,VBE respectively) have been argued in analogy with Fig. 2(f).

In ZGNRs [Fig. 2(d–g)] the effect of FM_*n* −*n*_ ordering is particularly rich, since the FM ordering contests the inherent AFM ordering of the edge states by favoring same spin at the two edges, similar(opposite) to that of the FM_*n* −*n*_ order if the biased region covers C-C zigzag(transverse) bonds. As shown schematically in Fig. 3, the contest supports the edge-states with spin-1 at edge-1, but suppresses the edge-state with spin-2 at edge-2. The consequent systematic closure of gap [Fig. 2(f,g)] exclusively for spin-2, [Fig. 3] can be understood as a result of effective increase in on-site energy for electrons with spin-2 at edge-2 due to accumulation of electrons with spin-1 at that edge on account of FM_*n* −*n*_. Expedient to recall, equal probability of finding an electron at both the edges, implying same effective on-site term at both the edges, leads to closure of band-gap, as happens for both the spins in the absence of Coulomb correlation. Indeed, the reduction in n-n FeM order near edge-2 due to the competing magnetic orders, implies reduction of the effective Coulomb correlation near edge-2 where spin-2 dominates. Similar effective reduction of Coulomb correlation should happens for both the spins as the biased region shifts towards the bulk, since it would increasingly lead to non-magnetic ground state and spin degenerate band-structure [Fig. 2(e)], akin to AGNRs. Nevertheless, with biased region covering C-C zigzag bonds [Fig. 2(f)], the inter-edge AFM ordering clearly evolves into inter-edge FM ordering, leading to evolution of the valence band edge for spin-2 into a partially occupied dispersive band, which offers a robust window for half-metallic transport. This reiterating that bias coverage of zigzag C atoms is more effective than that of biased transverse C atoms in emergence of FM_*n* −*n*_ order. However, the systematic reduction of band-gap for one of the spins, as argued above, happens in ZGNRs irrespective of whether the biased region covers C-C zigzag or transverse bonds, paving the way for robut bias controlled FM-semiconducting and half-metallic transport. Thus the same is offered by more realistic wider biased regions as well, at moderate *U* and low *V* ^*g*^, which might be technologically relevant.

Notably, the emergence of the half-metallic window in this case is rooted at the emergence of the FM_*n* −*n*_ order, and is thereby qualitatively different from previous proposals of bias induced opening of half-metallic window in ZGNRs where the sub-lattice asymmetry due to transverse electric field^13^ or defects^24,25^, lead to FM ordering beyond nearest neighbours in a ferrimagnetic ground state. Furthermore, the robust emergence of the FM_*n* −*n*_ order in the positively biased regions of the unit-cell, irrespective of location of the biased region with respect to the edges, and configuration of the ribbon edges, namely, zigzag or armchair or mix of both, clearly imply no explicit pre-requisition of flat or nearly flat bands at Fermi energy for the observed FM_*n* −*n*_ order to emerge. Thus the spatially non-uniform bias induced FM_*n* −*n*_ order proposed in this work is fundamentally different than the bias induced FM order^42^ and half-metallic transport^13,26^, reported so far.

### From first principles

**Figure 4:**
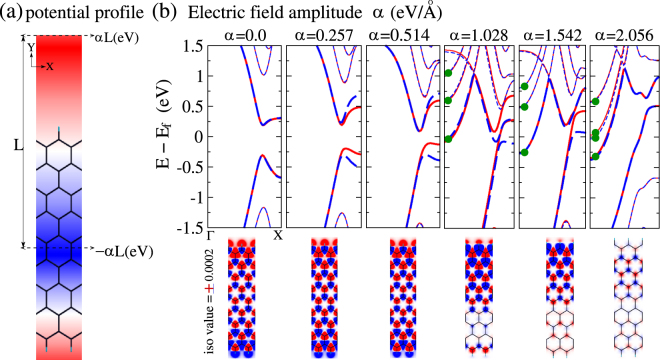
ZGNR in presence of sawtooth potential: (**a**) potential profile; (**b**) band structure (above) & spin density(below). DFT results are qualitatively similar to their counterparts [Fig. 2] based on mean-field Hubbard model. Bands marked by the green dots are exclusively due to space charge.

Band-structure calculated from first principles using density functional theory (DFT) with sawtooth potential^45^ applied in the transverse direction [Fig. 4(a)] of ZGNR to resemble biased region akin to those considered in Fig. 1, has similar trend as [Fig. 2(f)], marked by lifting of spin-degeneracy and reduction in band-gap for one of the spins [Fig. 4] within a range of ramp potential. This qualitative agreement between mean-field and DFT results is an important validation of the former, which only incorporates on-site Coulomb correlation. Furthermore, the reported agreement of DMRG, QMC and ED results^42,47^, with DFT in rationalizing FM ground state in doped AGNRs at moderate *U*, implies the confidence that our mean-field results will also be valid with improved consideration of correlation. Structural relaxation^48^ using forces derived from DFT ground states appear to indicate that the ribbons should maintain their structure intact in the range of ramp potential for which the FM_*n* −*n*_ order exists. However, in addition to the FM_*n* −*n*_ ordering, we also find highly dispersive free electron like bands around Fermi energy [Fig. 4(b)], whose origin is traced to accumulation of space charge between the periodic images of the ribbon. Upon emergence of FM_*n* −*n*_ order in the ribbon, the space charge also acquires a net non zero magnetic moment.

### Minimal model & mechanism

**Figure 5:**
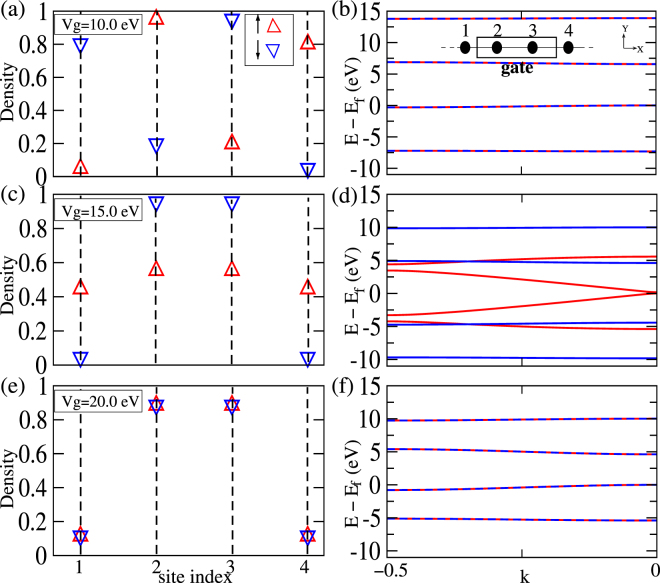
Density(up,down) and band structure of 4-site linear chain from mean-field Hubbard for *U* = 15.0:*V* ^*g*^ = 10.0 eV (**a**,**b**); *V* ^*g*^ = 15.0 eV (**c**,**d**); *V* ^*g*^ = 20.0 eV (**e**,**f**). The evolution of band structure is qualitatively similar to those in Fig. 2, implying generality of the FM_*n* −*n*_ order.

**Figure 6:**
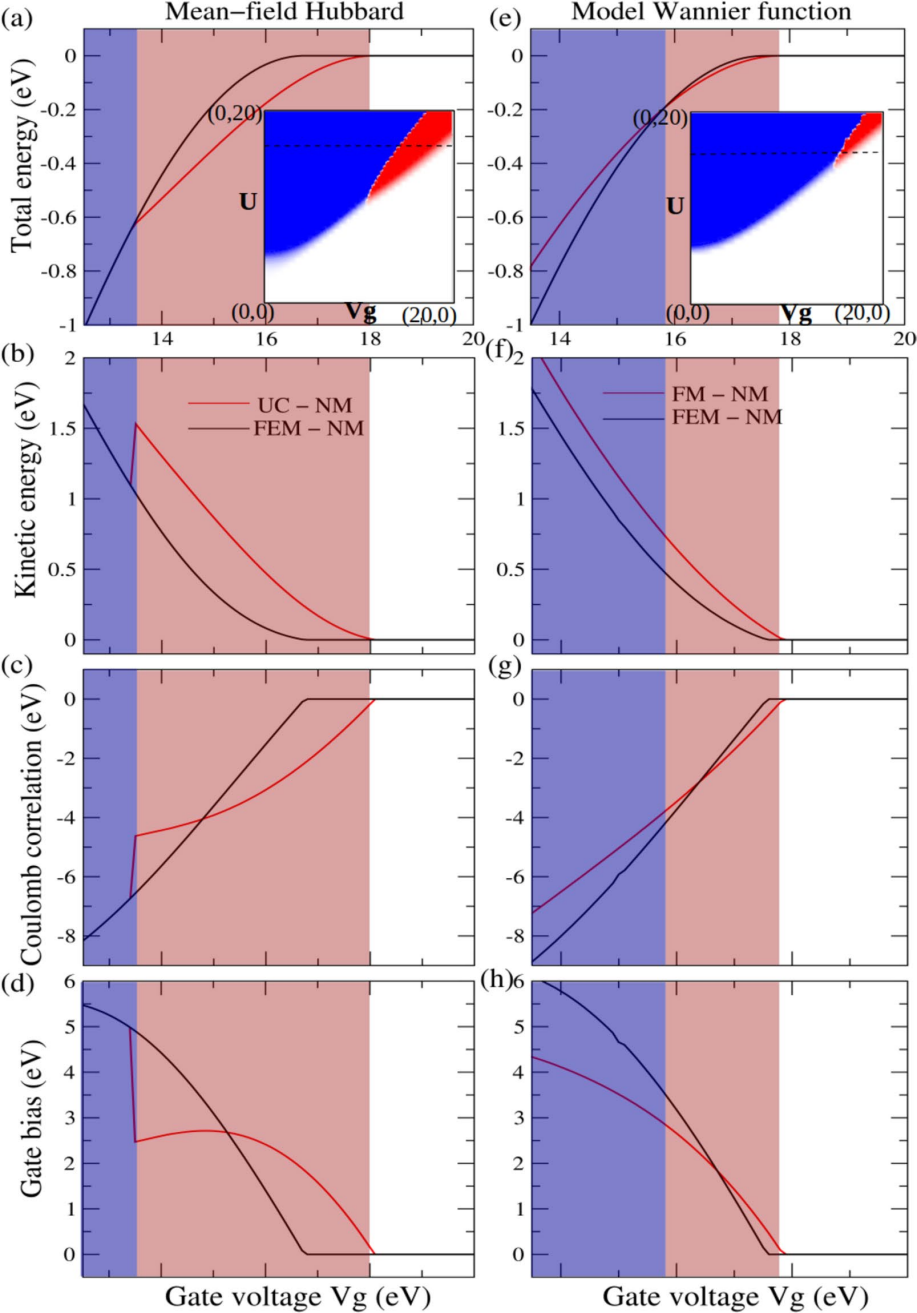
Biased site spin-correlation plot (inset) and different energy contributions for *U* = 15.0 from: mean-field Hubbard model(**a**–**d**) & analytic WF based calculation(**e**–**h**). The light red and blue backgrounds used in (**a**) to (**h**) represent FeM and FM ordering respectively between the biased sites. The qualitative similarity of the energetics responsible for emergence of the FM_*n* −*n*_ order, imply that the mean-field results are valid beyond the mean-field approximation.

To check the generality of our results beyond the three coordinated networks we resort to a minimal unit-cell, which can exhibit the FM_*n* −*n*_ order if it exists. We choose a unit-cell of four consecutive sites, of which two neighboring sites are biased [inset, Fig. 5(b)]. Spin-correlation between the two biased sites [inset, Fig. 6] as a function of *V* ^*g*^ and *U* has similar generic features as in Fig. 1. thus hinting at the generality of the n-n FM order as a property of non-uniformly biased bipartite systems. Notably, if we do not consider non-zero crystal momentum, then the positive spin-correlation does not exist, although the trend of spin-correlation as a function of *V* ^*g*^ and *U* obtained using the mean-field Hubbard model, agrees qualitatively with that obtained using exact-diagonalization(ED). Itinerant electrons described by dispersive bands at Fermi energy are thus likely to play important role in manifestation of FM_*n* −*n*_ order, as suggested by Fig. 2.

The evolution of spin polarized charge densities [Fig. 5(a,c,e)] with increasing *V* ^*g*^ suggests spin-separation between biased and unbiased region similar to that observed in ribbons. Such a spin-separation can possibly be rationalized by noting that increased occupancy of the biased sites by electrons with both the spins, upon increased *V* ^*g*^, would in turn increase potential energy due to on-site Coulomb repulsion. Thus to keep potential energy low, each of the biased sites will prefer to be dominated by electrons with one of the two spins. However if two such neighboring sites have opposite spins, as would be in a FeM ground state, then the wave-functions for both the spins will have rapid variation from site to site, leading to high kinetic energy. Instead, if wave-functions of one type of spin spans the biased sites more than those with the other spin, as evident from the charge densities [Fig. 5(a,c,e)], then the wave-functions can be smoother than their FeM counterparts, implying lesser kinetic energy while allowing lower on-site Coulomb repulsion as well. The evolution of charge densities [Fig. 5(a,c,e)] clearly implies electrons to be more itinerant for one spin than for the other upon emergence of FM_*n* −*n*_, leading to lifting of spin degeneracy [Fig. 5(c,d)] akin to that in [Fig. 2(a,c)]. Upon further increase of *V* ^*g*^ [Fig. 6(b–d)] the lowering of potential energy due to *V* ^*g*^ dominates over the increase of potential energy due to on-site Coulomb repulsion, leading to occupancy of bias sites by both spins, resulting into return of non-magnetic ground state and spin degenerate band-structure [Fig. 5(e,f)]. Thus with higher *U* a higher *V* ^*g*^ is required for the FM_*n* −*n*_ order to quench, which is also consistent with the trend observed in Fig. 1.

To quantitatively justify the mechanism anticipated above, we partition the total energy [Fig. 6(a)] of the unconstrained(UC) ground state into kinetic energy and potential energies due to on-site Coulomb repulsion and applied bias, and compare those with their counterparts in a non-magnetic(NM) and FeM ground states. NM ground state is obtained by assigning same charge density for both the spins. For the FeM ground state, charge density for one of the spins is assigned to be the mirror image of that for the other spin about the centre of the unit-cell. We choose the energetics of the NM ground state to be the reference. Figure 6(c,d) suggests that the UC ground state with FM_*n* −*n*_ order has lower energy than NM and FeM ground states, owing initially to lowering of potential energy due to gate bias, but subsequently and primarily due to lowering of on-site Coulomb repulsion facilitated by separation of spin between positively biased and unbiased sites. Notably, although the FeM ground state has a lower kinetic energy than the UC ground state, the degree of localization at the positively biased site offered by the former is much lower than that due to the latter. Thus an FeM ordered state with the same degree of localization as offered by the UC ground state must have higher kinetic energy than the latter. Thus the observed FM_*n* −*n*_ order is a result of attempts to minimize on-site Coulomb repulsion and kinetic energy in conjunction with each other, while maximizing localization of electrons at the positively biases sites.

In addition to agreement with DFT results, to demonstrate further that the observed FM_*n* −*n*_ order is not limited by the mean-field approximation, we resort to simple analytical models for Wannier functions(WF), to represent the ground state of the four site unit-cell at half-filling. WFs are linear combination of wave-functions and can be chosen to be real and localized largely within a unit-cell in one dimension. Thus in place of 2*N*_*k*_ wave-functions for each spin, *N*_*k*_ being the number of allowed wave-vectors in the first Brillouin zone, we can choose two WFs for each spin to represent four electrons. We approximate WFs to be non-zero only within a unit-cell. Such approximate WFs describing the non-magnetic(NM) ground state can be of the following general form:

$$\begin{array}{rcl}{\varphi }_{\mathrm{1,}\uparrow /\downarrow }^{NM} & = & (a,b,c,d),\\ {\varphi }_{\mathrm{2,}\uparrow /\downarrow }^{NM} & = & (e+\frac{c\mathrm{.}f}{a},g+\frac{d\mathrm{.}f}{b},-f-\frac{a\mathrm{.}e}{c},-f-\frac{g\mathrm{.}b}{d}),\end{array}$$

which are constrained to be orthogonal to each other. Similarly, two orthogonal WFs to describe the FeM ground state can be approximated as:

$$\begin{array}{rcl}{\varphi }_{\mathrm{1,}\uparrow }^{FeM} & = & (a,b,c,d),\\ {\varphi }_{\mathrm{2,}\uparrow }^{FeM} & = & (e+\frac{c\mathrm{.}f}{a},g+\frac{d\mathrm{.}f}{b},-f-\frac{a\mathrm{.}e}{c},-f-\frac{g\mathrm{.}b}{d}),\\ {\varphi }_{\mathrm{1,}\downarrow }^{FeM} & = & (d,c,b,a),\\ {\varphi }_{\mathrm{2,}\downarrow }^{FeM} & = & (f+\frac{g\mathrm{.}b}{d},f+\frac{a\mathrm{.}e}{c},-g-\frac{d\mathrm{.}f}{b},-e-\frac{c\mathrm{.}f}{a}),\end{array}$$

where the |*ϕ*_*i*,↑_|^2^ is mirror image of |*ϕ*_*i*,↓_|^2^ with respect to the middle of the unit-cell. Finally, orthogonal WFs with FM_*n* −*n*_ order can be approximated as:

$$\begin{array}{rcl}{\varphi }_{\mathrm{1,}\uparrow }^{FM} & = & (a,b,b,a),\\ {\varphi }_{2\uparrow }^{FM} & = & (c,d,-d,-c),\\ {\varphi }_{1\downarrow }^{FM} & = & (e,f,f,e),\\ {\varphi }_{2\downarrow }^{FM} & = & g,h,-h,-g.\end{array}$$

The number of independent variables chosen to express the WFs are determined by the symmetry of the spin densities [Fig. 5(a,c,e)] and orthogonality of the states. Total energies of ground states are calculated in the basis of the approximate WFs within the Hubbard model without mean-field approximation. For each class of WFs, ground state is obtained by finding the global minima of total energy using the cylindrical algebraic decomposition algorithm^49^. Kinetic energy and potential energies due to Coulomb repulsion and external bias are estimated using the WFs corresponding to the ground states. Notably, Fig. 6(e–h) implies emergence of FM_*n* −*n*_ order in exactly the same pathway as suggested within the mean-field approximation of Hubbard model [Fig. 6(a–d)]. These agreements indeed confirms the induced FM_*n* −*n*_ order to be a genuine attribute of in-homogeneously biased bipartite systems and should be observed beyond mean-field computation, as also suggested by DFT. Thus the FM_*n* −*n*_ order proposed in this work indeed appears to be a general property of non-uniformly biased bipartite systems. Although it remains to be specifically tested, available studies^44^ suggest that similar emergence of FM_*n* −*n*_ order may be even easier in non-bipartite systems.

## Conclusion

To summarize, in this work we computationally propose the possibility of bias driven nearest neighbour(n-n) ferromagnetic(FM) order and half-metallic transport as a result of interplay of localization and Coulomb correlation. Results suggest emergence of n-n FM order in positively biased regions of non-uniformly biased bipartite systems, as a generic outcome of efforts to minimize Coulomb repulsion with minimal loss of the itinerant nature of electrons, while maximizing localization of electrons in the positively biased sites. The n-n FM order is computationally demonstrated here to exist in graphene nano-ribbons irrespective of their edge configurations, as well as in a minimal model system, which emphasizes the generality of the observed localization induced n-n FM order at half filling. The associated lifting of spin-degeneracy leads to exotic metallic (normal, ferromagnetic and half-metallic) phases. In armchair edged semiconducting ribbons the metallic phases are presided by direct to indirect transition, while in zigzag edged graphene ribbons, their inherent inter-edge AFM order contests the bias driven FM_*n* −*n*_ order, leading to systematic closer of gap for one of the spins resulting into robust window for FM semiconducting and half-metallic transport. These results are expected to encourage a conceptually new pathway for voltage controlled opening of windows for half-metallic transport in two dimensional systems in general. In view of recent advancements^50–52^, in implementing gates at sub-micron length-scale, these results might encourage implementation of the proposed pathway for voltage controlled systematic opening of windows for half-metallic transport in ribbons and sheets of graphene and other two dimensional systems.

## Method

To compute spin-polarized electronic structure in the realistic AGNR and ZGNR unit-cells, we resort to the mean-field approximation of Hubbard model within the n-n tight-binding framework:

2 $$H=-t(\sum _{\langle i,j\rangle ,\sigma }\,{c}_{i,\sigma }^{\dagger }{c}_{j,\sigma }+h\mathrm{.}c)+\sum _{i,\sigma }\,{c}_{i,\sigma }^{\dagger }{c}_{i,\sigma }(U\langle {n}_{i,\sigma ^{\prime} }\rangle -{V}_{i}^{g}+{V}_{i}^{q})$$

〈*n*_*i*,*σ*′_〉 being the population of electron with spin-*σ* at the *i*-th site due to the occupied states. Eqn. (2) implies a self-consistent computation of electronic structure as a function of the strength of on-site Coulomb repulsion (*U*)^53,54^, between opposite spins, and the gate bias $\{{V}_{i}^{g}\}$, given the lowering of energy due to hopping between n-n sites represented by *t* = −2.7 *eV* ^55–57^. Coulomb potential (*V* ^*q*^) due to electrons at nearest neighboring sites and beyond is calculated using the Ewald summation scheme^58^. We compare our mean-field Hubbard model based results with their counterparts obtained from first principles using density functional theory (DFT). We use a plane-wave based implementation^45^ of DFT, wherein, we have used a gradient corrected functional^59^ of density to approximately estimate the exchange-correlation contribution to total energy.

Standard computational techniques have been used in this work to calculate electronic structure of systems detailed in the figures.

## References

[1] Son Y-W, Cohen ML, Louie SG (2006). Energy gaps in graphene nanoribbons. Phys. Rev. Lett..

[2] Castro Neto AH, Guinea F, Peres NMR, Novoselov KS, Geim AK (2009). The electronic properties of graphene. Rev. Mod. Phys..

[3] Avouris P, Chen Z, Perebeinos V (2007). Carbon-based electronics. Nat. Nanotechnol..

[4] Yazyev OV (2010). Emergence of magnetism in graphene materials and nanostructures. Reports on Progress in Physics.

[5] Lieb EH (1989). Two theorems on the Hubbard model. Phys. Rev. Lett..

[6] Han W, Kawakami RK, Gmitra M, Fabian J (2014). Graphene spintronics. Nat Nano..

[7] Li X, Wang X, Zhang L (2008). Chemically Derived, Ultrasmooth Graphene Nanoribbon Semiconductors. Science.

[8] Ezawa M (2006). Electronic properties of carbon nanoribbons and peculiar width dependence. Phys. Rev. B.

[9] Sun L, Li Q, Ren H, Su H, Shi QW (2008). Strain effect on electronic structures of graphene nanoribbons: A first-principles study. The Journal of Chemical Physics.

[10] Rozhkov AV (2011). Electronic properties of mesoscopic graphene structures: Charge confinement and control of spin and charge transport. Physics Reports..

[11] Esquinazi P (2003). Induced Magnetic Ordering by Proton Irradiation in Graphite. Phys. Rev. Lett..

[12] Lehtinen PO, Foster AS, Ma Y, Krasheninnikov AV, Nieminen RM (2004). Irradiation-Induced Magnetism in Graphite: A Density Functional Study. Phys. Rev. Lett..

[13] Son Y-W, Cohen ML, Louie SG (2006). Half-Metallic Graphene Nanoribbons. Nature (London).

[14] Raza H, Kan E (2008). Armchair graphene nanoribbons: Electronic structure and electric-field modulation. Phys. Rev. B.

[15] Yazyev OV, Helm L (2007). Defect-induced magnetism in graphene. Phys. Rev. B.

[16] Boukhvalov DW, Katsnelson MI (2008). Chemical functionalization of graphene with defects. Nano Letters.

[17] Nair, R. R., Tsai, I.-L. & Sepioni, M. Dual origin of defect magnetism in graphene and its reversible switching by molecular doping. *Nature Communications* **4** (2013). 10.1038/ncomms3010 23760522

[18] Santos EJG (2008). Switching on magnetism in Ni-doped graphene: Density functional calculations. Phys. Rev. B.

[19] Cervantes-Sodi F, Csányi G, Piscanec S, Ferrari AC (2008). Edge-functionalized and substitutionally doped graphene nanoribbons: Electronic and spin properties. Phys. Rev. B.

[20] Deng X, Wu Y, Dai J (2011). Electronic structure tuning and band gap opening of graphene by hole/electron codoping. Physics Letters A.

[21] Dai XQ (2011). First-principle study of magnetism induced by vacancies in graphene. Eur. Phys. J. B.

[22] Yan L (2012). Chemistry and physics of a single atomic layer: strategies and challenges for functionalization of graphene and graphene-based materials. Chem. Soc. Rev..

[23] Dutta S, Pati SK (2008). Half-Metallicity in Undoped and Boron Doped Graphene Nanoribbons in the Presence of Semilocal Exchange-Correlation Interactions. J. Phys. Chem. B.

[24] Kan EJ, Li Z, Yang J, Hou JG (2008). Half-Metallicity in Edge-Modified Zigzag Graphene Nanoribbons. J. Am. Chem. Soc..

[25] Bhattacharjee J (2012). Half-metallicity in graphene nanoribbons with topological defects at edge. J. Chem. Phys..

[26] Zhang WX (2014). Voltage-driven spintronic logic gates in graphene nanoribbons. Scientific Reports.

[27] Ritter Kyle A, Lyding, Joseph W (2009). The influence of edge structure on the electronic properties of graphene quantum dots and nanoribbons. Nature Materials.

[28] Magda G (2014). Room-temperature magnetic order on zigzag edges of narrow graphene nanoribbons. Nature.

[29] Mott NF (1949). The Basis of the Electron Theory of Metals, with Special Reference to the TransitionMetals. Proc. Phys. Soc. London, Ser. A.

[30] Martelo LM, Dzierzawa M, Siffert L, Baeriswy D (1997). Mott-Hubbard transition and antiferromagnetism on the honeycomb lattice. Z. Phys. B.

[31] Fazekas, P. *Series in Modern Condensed Matter Physics* - Vol. 5 {5 Toh Tuck Link, Singapore 596224,World Scientific Publishing Co. Pte. Ltd.} (1999).

[32] Soriano D, Fernández-Rossier J (2012). Interplay between sublattice and spin symmetry breaking in graphene. Phys. Rev. B.

[33] Lin H, Fratesi G, Brivio GP (2015). Graphene magnetism induced by covalent adsorption of aromatic radicals. Phys. Chem. Chem. Phys..

[34] Jung J, MacDonald AH (2009). Carrier density and magnetism in graphene zigzag nanoribbons. Phys. Rev. B.

[35] Dutta S, Wakabayashi K (2012). Tuning charge and spin excitations in zigzag edge nanographene ribbons. Sci. Rep..

[36] Hubbard J (1963). Electron Correlations in Narrow Energy Bands. Proc. R. Soc. London A.

[37] Amadon JC, Hirsch JE (1996). Metallic ferromagnetism in a single-band model: Effect of band filling and Coulomb interactions. Phys. Rev. B.

[38] Nagaoka Y (1966). Ferromagnetism in a narrow, almost half-filled s band. Phys. Rev..

[39] Tasaki H (1989). Extension of Nagaoka’s theorem on the large-U Hubbard model. Phys. Rev. B.

[40] Mielke A, Tasaki H (1993). H. Ferromagnetism in the Hubbard model. Examples from models with degenerate single-electron ground states. Commun. Math. Phys..

[41] Tasaki H (1998). From Nagaoka’s Ferromagnetism to Flat-Band Ferromagnetism and Beyond: An Introduction to Ferromagnetism in the Hubbard Model. Prog. Theor. Phys..

[42] Lin H-H, Hikihara T, Jeng H-T (2009). Ferromagnetism in armchair graphene nanoribbons. Phys. Rev. B.

[43] Stoner E (1938). Collective Electron Ferromagnetism. Proc. R. Soc. London, Ser. A.

[44] Kan M (2012). Tuning magnetic properties of graphene nanoribbons with topological line defects: From antiferromagnetic to ferromagnetic. Phys. Rev. B.

[45] Giannozzi, P. *et al*. Quantum Espresso: A Modular and Open-source Software Project for Quantum Simulations of Materials. *J. Phys. Cond. Mat*. **21** 395502(1–20) (2009). 10.1088/0953-8984/21/39/395502 21832390

[46] Sorella S, Tosatti E (1992). Semi-Metal-Insulator Transition of the Hubbard Model in the Honeycomb Lattice. Europhys. Lett..

[47] Feldner H (2010). Magnetism of finite graphene samples: Mean-field theory compared with exact diagonalization and quantum Monte Carlo simulations. Phys. Rev. B.

[48] Fletcher, R. Practical Methods of Optimization; Wiley: New York; (1987).

[49] Wolfram Research, Inc., Mathematica, Version 10.0, Champaign, IL (2014).

[50] Liao L (2010). High- *k* oxide nanoribbons as gate dielectrics for high mobility top-gated graphene transistors. Proc Natl Acad Sci. USA.

[51] Liao L (2010). High-speed graphene transistors with a self-aligned nanowire gate. Nature.

[52] Wu Y (2011). High-frequency, scaled graphene transistors on diamond-like carbon. Nature.

[53] Baeriswyl D, Maki K (1985). Electron correlations in polyacetylene. Phys. Rev. B.

[54] Jeckelmann E, Baeriswyl D (1994). The metal-insulator transition in polyacetylene: variational study of the Peierls-Hubbard model. Synth. Met..

[55] Mintmire JW, Dunlap BI, White CT (1992). Are fullerene tubules metallic?. Phys. Rev. Lett..

[56] Wilder JWG, Venema LC, Rinzler AG, Smalley RE, Dekker C (1998). Electronic structure of atomically resolved carbon nanotubes. Nature (London).

[57] Odom TW, Huang JL, Kim P, Lieber CM (1998). Atomic structure and electronic properties of single-walled carbon nanotubes. Nature (London).

[58] Bródka A (2002). Ewald type summation method for electrostatic interactions in computer simulations of a three-dimensional system periodic in one direction. Chemical Physics Letters.

[59] Perdew JP, Burke K, Ernzerhof M (1996). Generalized Gradient Approximation Made Simple. Phys. Rev. Lett.

